# Detection of Novel Orthobunyavirus Reassortants in Fatal Neurologic Case in Horse and *Culicoides* Biting Midges, South Africa

**DOI:** 10.3201/eid3107.241800

**Published:** 2025-07

**Authors:** Matshepo Elizabeth Rakaki, Miné van der Walt, June Williams, Marietjie Venter

**Affiliations:** University of Pretoria, Pretoria, South Africa (M.E. Rakaki, J. Williams, M. Venter); University of the Witwatersrand, Johannesburg, South Africa (M. van der Walt, M. Venter)

**Keywords:** *Orthobunyavirus*, orthobunyaviruses, Shuni virus, Shamonda virus, Caimito virus, Sango virus, reassortants, *Culicoides* biting midges, horse, vector-borne infections, viruses, zoonoses, South Africa

## Abstract

We detected Shuni virus in horses and ovine fetuses and Shamonda virus in a caprine fetus in South Africa. We identified a Shuni/Shamonda virus reassortant in a horse and Shuni/Caimito, Shamonda/Caimito, and Shamonda/Sango virus reassortants in *Culicoides* midges. Continued genomic surveillance will be needed to detect orthobunyavirus infections in Africa.

The *Orthobunyavirus* genus, in the family Peribunyaviridae, has 18 serogroups; several of those represent emerging or reemerging viruses of importance to human and animal health transmitted by mosquitoes and *Culicoides* biting midges (*1*). Orthobunyavirus genomes have 3 negative single-stranded RNA segments, small (S), medium (M), and large (L) (*2*). Those segments can reassort in nature (*3*,*4*), leading to genetically diverse viruses with altered host ranges and pathogenicity (*3*). We characterized the genomes of orthobunyaviruses detected in animals with neurologic signs and birth defects and in *Culicoides* biting midges isolated in South Africa.

## The Study

During January 2020–February 2023, samples from 280 equids, 35 ruminants, 53 wildlife species, 6 avians species, and 7 cats or dogs displaying unexplained febrile and neurologic signs or sudden unexplained death were submitted from across South Africa to the zoonotic arbovirus surveillance program at the University of Pretoria (Pretoria, South Africa). We extracted RNA from blood, serum, plasma, cerebrospinal fluid, or tissue samples in a Biosafety Level 3 laboratory by using the QIAamp Viral RNA Mini Kit or RNeasy Mini Kit (QIAGEN, https://www.qiagen.com). We screened animal samples by using in-house S segment–specific TaqMan real-time reverse transcription PCR (RT-PCR) for *Orthobunyavirus* Simbo serogroup, as previously described (*5*). We characterized the M and L segments of positive samples by using conventional nested PCR. Primers for the M segment specifically targeted orthobunyaviruses (*6*), whereas L segment primers targeted the Peribunyaviridae family (*7*) (Appendix 1 Table 1).

A total of 6/381 (1.6%) animals tested positive for orthobunyaviruses by PCR of the S segment (Table; Appendix 1 Table 2) (*5*,*8*). Sanger sequencing identified 5/6 (83.3%) viruses as Shuni virus (SHUV); 4/5 (80.0%) infections were fatal. We found SHUV in samples from 2 aborted ovine fetuses from Mpumalanga and Northwest Provinces and 3 horses from Gauteng and the Northern Cape Provinces. An orthobunyavirus detected in an aborted goat fetus from the Western Cape Province was identified as Shamonda virus (SHAV) through Sanger sequencing, as previously described (*6*). We attempted next-generation sequencing but were unable to obtain full genomes.

**Table:** Orthobunyaviruses identified by PCR and sequencing of S, M, and L segments in samples from mammals and *Culicoides* biting midges, South Africa*

Sample no.	Orthobunyavirus RT-PCR S segment	Conventional nested PCR		
		S segment	M segment	L segment
Mammals				
ZRU053/20	Shuni virus	IS	IS	IS
ZRU012/21	Shuni virus	Shuni virus	Shamonda virus	Shamonda virus
ZRU027/21	Shuni virus	AF	AF	AF
ZRU093/21	Shamonda virus	Shamonda virus	Shamonda virus	Shamonda virus
ZRU019/22	Shuni virus	Shuni virus	AF	AF
ZRU099/22	Shuni virus	Shuni virus	AF	Shuni virus
*Culicoides* biting midges				
MAR538_16	Schmallenberg virus	Schmallenberg virus	Schmallenberg virus	Schmallenberg virus
MAR085_13	Sabo virus	AF	Sabo virus	AF
MAR032_12	Sango virus	AF	Shamonda virus	AF
KYA229_16	Shuni virus	Shuni virus	AF	Shuni virus
GAU272_17	Shamonda virus	AF	AF	Caimito virus
GAU110_14	Shamonda virus	AF	AF	Caimito virus
MAR057_13	Shuni virus	AF	AF	Caimito virus

*AF, amplification failed; IS, insufficient sample; RT-PCR, reverse transcription PCR.

**Figure 1:**
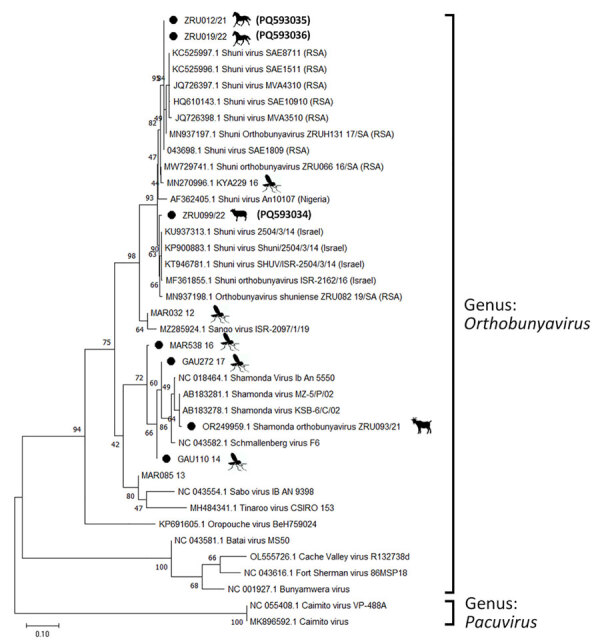
Phylogenetic analysis of *Orthobunyavirus* S segments in study of reassortants in fatal neurologic case in horse and *Culicoides* biting midges, South Africa. Tree shows phylogenetic analysis of positive animal cases and *Culicoides* pools for the S segment of sequences from South Africa (black dots) relative to orthobunyavirus reference strains available in GenBank within the Simbu serogroup. Tree was constructed in MEGA X (https://www.megasoftware.net) by using the Tamura 3-parameter model. Bootstrap values from 1,000 replicates are shown at each node. *Pacuvirus* genus was included as an outgroup to root the tree. Bolded accession numbers in parentheses indicate sequences from this study that were submitted to GenBank. Scale bar indicates nucleotide substitutions per site.

**Figure 2:**
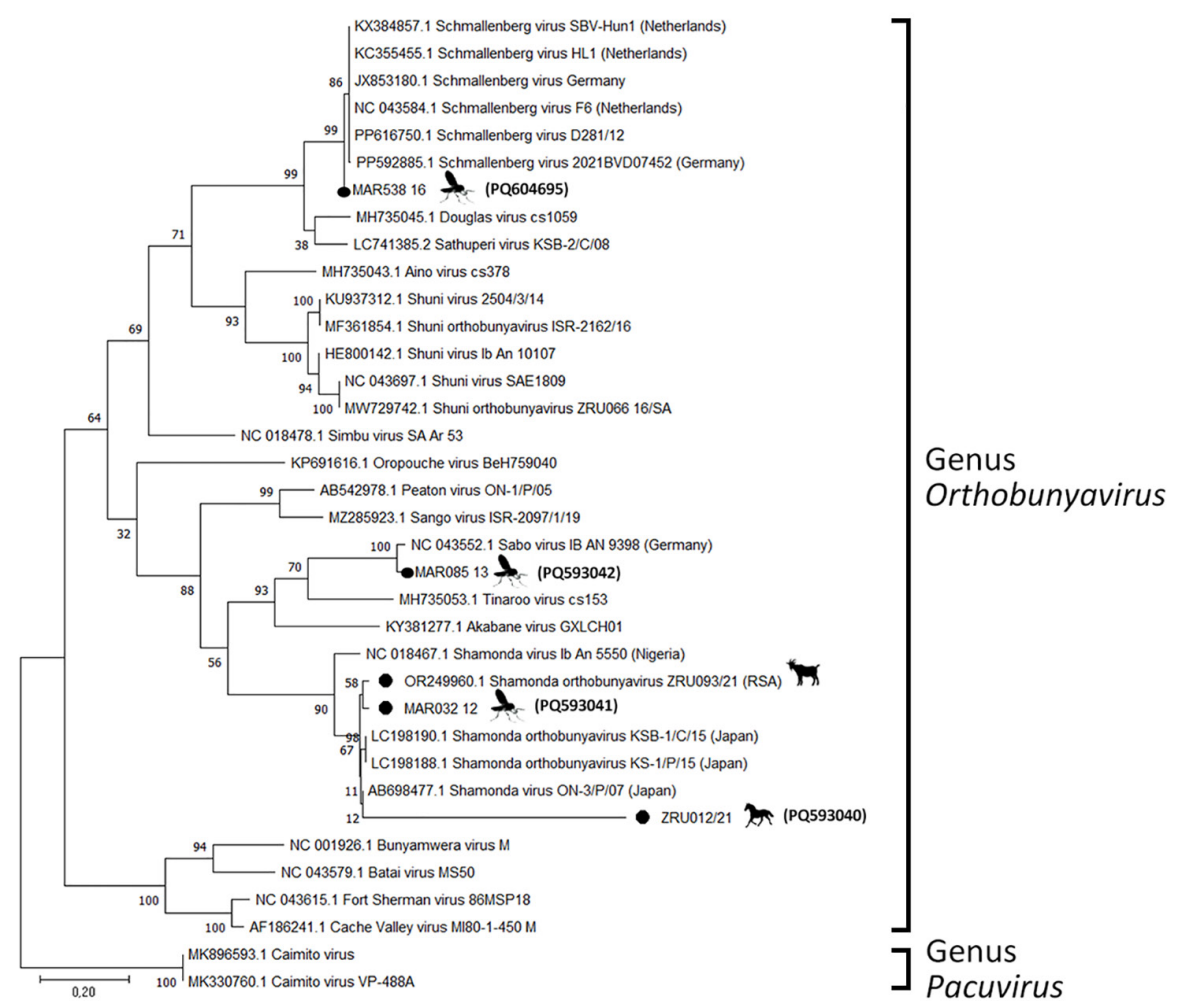
Phylogenetic analysis of *Orthobunyavirus* M segments in study of reassortants in fatal neurologic case in horse and *Culicoides* biting midges, South Africa. Tree shows phylogenetic analysis of the positive animal cases and *Culicoides* pools for the M segment of sequences from South Africa (black dots) relative to orthobunyavirus reference strains available in GenBank within the Simbu serogroup. Tree was constructed in MEGA X (https://www.megasoftware.net) by using the Hasegawa-Kishino-Yano model. Bootstrap values from 1,000 replicates are shown at each node. *Pacuvirus* genus was included as an outgroup to root the tree. Bolded accession numbers in parentheses indicate sequences from this study that were submitted to GenBank. Scale bar indicates nucleotide substitutions per site.

**Figure 3:**
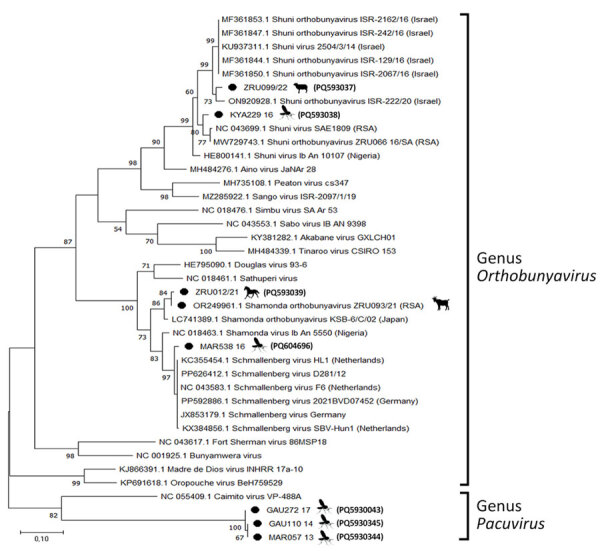
Phylogenetic analysis of *Orthobunyavirus* L segments in study of reassortants in fatal neurologic case in horse and *Culicoides* biting midges, South Africa. Tree shows phylogenetic analysis of the positive animal cases and *Culicoides* pools for the L segment of sequences from South Africa (black dots) relative to orthobunyavirus reference strains available in GenBank within the Simbu serogroup. Tree was constructed in MEGA X (https://www.megasoftware.net) by using the Tamura-Nei model. Bootstrap values from 1,000 replicates are shown at each node. *Pacuvirus* genus was included as an outgroup to root the tree. Bolded accession numbers in parentheses indicate sequences from this study that were submitted to GenBank. Scale bar indicates nucleotide substitutions per site.

We determined pairwise distances between orthobunyavirus sequences and constructed maximum-likelihood phylogenetic trees by using MEGA X version 10.2.6 (https://www.megasoftware.net) (Appendix 2 Tables 1–4). We performed conventional RT-PCR to obtain a larger S segment fragment (291 bp) and successfully amplified 4 (66.7%) of 6 sequences (Figure 1). The ZRU099/22 (ovine fetus) S segment grouped with SHUV strains from Israel (97.0%–98.0% identity) (Figure 1). Two horse samples (ZRU12/22 and ZRU19/22) grouped with SHUV strains from South Africa (97.0%–100.0% identity). The S segment of the aborted goat sample (ZRU93/21) clustered with SHAV (*6*). We amplified M segments for 2 (33.3%) of 6 animal samples. Both M segments of ZRU093/21 (*6*) and ZRU012/21 (detected in horse brain) clustered with SHAV (Figure 2); the S segment of ZRU012/21 also clustered with SHUV. The M segment of ZRU012/21 had 63.0%–65.0% identity with SHAV. We amplified L segments of 3/6 (50.0%) animal samples. Of those, 2 samples grouped with SHAV, including the aborted goat fetus (ZRU093/21) (*6*) and horse sample (ZRU012/21) (Figure 3), suggesting ZRU012/21 is a reassortment of the SHUV S segment and SHAV M and L segments; the L segment had 96.0%–99.0% identity with SHAV. The third sample (ZRU099/22) from the ovine fetus clustered with SHUV and had 96% identity with strains from Israel and 92% identity with strains from South Africa.

To determine if reassortants could be identified in *Culicoides* midges previously identified as orthobunyavirus positive, we screened pools of midges collected from 6 surveillance sites in Mpumalanga, Limpopo, and Gauteng Provinces during 2013–2017 (*9*). We performed M and L segment–specific RT-PCR on RNA from 7 orthobunyavirus S segment–positive *Culicoides* pools previously characterized by Sanger sequencing (Table; Figure 1). We successfully amplified the M segment from 3 pooled samples, all from Marakele National Park in Limpopo Province. MAR538_16 grouped with the Schmallenberg virus (SBV) M segment (97.0%–98.0% identity) (Figure 2), as well as the SBV S segment (*9* (Table). MAR085_13, previously positive for the Sabo virus (SABOV) S segment, also grouped with the SABOV M segment (95.0% identity). MAR032_12 grouped with the SHAV M segment (97.0% identity) but also grouped with the Sango virus (SANV) S segment, suggesting reassortment. We amplified L segments for 5 pooled *Culicoides* midge samples: 2 from Marakele National Park in Limpopo Province, 2 from Boschkop in Pretoria Province, and 1 from Kyalami in Midrand, Gauteng Province. KYA299_16 grouped with L segments of SHUV strains from South Africa (97.0% identity) (Figure 3) and SHUV S segments. MAR538_16 grouped with SBV L segments (97.0%–98.0% identity) and both S and M segments. GAU272_17 and GAU110_14 grouped with the Caimito virus (CAIV) L segment (61.0% identity); however, both grouped with SHAV S segments, suggesting a possible reassortment. MAR057_13 also grouped with the CAIV L segment (59.0% identity) (Figure 3) but grouped with SHUV S segments, suggesting another possible reassortment. We summarized the orthobunyavirus-positive cases tested in this study (Table). We deposited sequences in GenBank (accession numbers in Figures 1–3).

SHUV has been circulating in South Africa in both *Culicoides* midge and mosquito vectors and has been detected in wildlife, ruminants, horses, birds, and, more recently, in humans (*5*,*8*–*12*). SHAV was first detected in *Culicoides* biting midges in Gauteng Province of South Africa in 2017 and in an aborted goat fetus from the Western Cape in 2021 (*6*,*9*). SHUV was detected in 3 horses with neurologic signs, 2 from Gauteng and 2 from the Western Cape during 2020–2022. One of the 2 fatal cases was caused by a novel SHUV/SHAV reassortment. SHUV was also detected in 2 aborted ovine fetuses. Abortions associated with SHUV have been described in ruminants in Israel. We previously reported SHUV in cerebrospinal fluid from a 13-day-old, hospitalized infant with hydrops fetalis in Gauteng Province (*12*), suggesting SHUV might be a risk factor for birth defects in humans. SHUV, SHAV, and SBV, as well as reassortants, were detected in animals and *Culicoides* midges in this study. Therefore, orthobunyavirus infections should be considered a possible causes of birth defects in humans and animals in Africa. 

Reassortments might alter pathogenesis, which has been observed for Ngari virus (*13*,*14*). Reassortants were detected in *Culicoides* midges collected in Gauteng and Limpopo Provinces; 2 were SHAV/CAIV, 1 SHUV/CAIV, and 1 SANV/SHAV reassortants. SANV was previously detected in a springbok that displayed neurologic signs in Mpumalanga Province in South Africa (*5*). Little is known about CAIV, which belongs to the genus *Pacuvirus* isolated in Brazil from rodents and sandflies (*15*). The M segment of orthobunyaviruses detected in South Africa had only 61% identity to CAIV viruses from South America, suggesting different virus species within that genus.

The first limitation of our study is that full genome sequencing was not successful for mammal and *Culicoides* samples, likely because of low RNA levels. We were also unable to reamplify all samples, suggesting RNA degradation in older samples.

## Conclusions

Co-circulation of several orthobunyaviruses in Africa might give rise to novel reassortants with altered host range, tissue tropism, and pathogenesis. Reassortants might be missed without characterization of all 3 virus segments. A SHUV/SHAV reassortant described in this study was associated with severe neurologic infection and death in a horse, and SHUV and SHAV were both associated with abortions in ruminants. Several orthobunyaviruses were detected in *Culicoides* midges, including variants clustering with SBV and reassortants involving SHUV, SHAV, SANV, and a CAIV-like virus. Continued One Health genomic surveillance will be needed to detect those and other orthobunyaviruses to determine risks for infections in animals and humans in Africa and elsewhere.

Appendix 1 Additional information for detection of novel orthobunyavirus reassortants in fatal neurologic case in horse and *Culicoides* biting midges, South Africa.

Appendix 2 Additional information for pairwise distances calculated for S, M, and L segments between orthobunyaviruses from this study and GenBank reference strains.

## References

[1] Briese T, Calisher CH, Higgs S. Viruses of the family Bunyaviridae: are all available isolates reassortants? Virology. 2013;446:207–16. 10.1016/j.virol.2013.07.030 24074583

[2] Elliott RM. Orthobunyaviruses: recent genetic and structural insights. Nat Rev Microbiol. 2014;12:673–85. 10.1038/nrmicro3332 25198140

[3] Heitmann A, Gusmag F, Rathjens MG, Maurer M, Frankze K, Schicht S, et al. Mammals preferred: reassortment of *Batai* and *Bunyamwera orthobunyavirus* occurs in mammalian but not insect cells. Viruses. 2021;13:1702. 10.3390/v13091702 34578285 PMC8473249

[4] Yanase T, Aizawa M, Kato T, Yamakawa M, Shirafuji H, Tsuda T. Genetic characterization of Aino and Peaton virus field isolates reveals a genetic reassortment between these viruses in nature. Virus Res. 2010;153:1–7. 10.1016/j.virusres.2010.06.020 20600386

[5] Steyn J, Motlou P, van Eeden C, Pretorius M, Stivaktas VI, Williams J, et al. Shuni virus in wildlife and nonequine domestic animals, South Africa. Emerg Infect Dis. 2020;26:1521–5. 10.3201/eid2607.190770 32568048 PMC7323521

[6] Walt MV, Rakaki ME, MacIntyre C, Mendes A, Junglen S, Theron C, et al. Identification and molecular characterization of Shamonda virus in an aborted goat fetus in South Africa. Pathogens. 2023;12:1100. 10.3390/pathogens12091100 37764908 PMC10536486

[7] Kopp A, Hübner A, Zirkel F, Hobelsberger D, Estrada A, Jordan I, et al. Detection of two highly diverse peribunyaviruses in mosquitoes from Palenque, Mexico. Viruses. 2019;11:832. 10.3390/v11090832 31500304 PMC6783978

[8] Motlou TP, Williams J, Venter M. Epidemiology of Shuni virus in horses in South Africa. Viruses. 2021;13:937. 10.3390/v13050937 34069356 PMC8158722

[9] Snyman J, Venter GJ, Venter M. An investigation of *Culicoides* (Diptera: Ceratopogonidae) as potential vectors of medically and veterinary important arboviruses in South Africa. Viruses. 2021;13:1978. 10.3390/v13101978 34696407 PMC8541229

[10] van Eeden C, Williams JH, Gerdes TG, van Wilpe E, Viljoen A, Swanepoel R, et al. Shuni virus as cause of neurologic disease in horses. Emerg Infect Dis. 2012;18:318–21. 10.3201/eid1802.111403 22305525 PMC3310469

[11] Guarido MM, Motlou T, Riddin MA, MacIntyre C, Manyana SC, Johnson T, et al. Potential mosquito vectors for Shuni virus, South Africa, 2014–2018. Emerg Infect Dis. 2021;27:3142–6. 10.3201/eid2712.203426 34808093 PMC8632193

[12] Motlou TP, Venter M. Shuni virus in cases of neurologic disease in humans, South Africa. Emerg Infect Dis. 2021;27:565–9. 10.3201/eid2702.191551 33496223 PMC7853583

[13] Gerrard SR, Li L, Barrett AD, Nichol ST. Ngari virus is a Bunyamwera virus reassortant that can be associated with large outbreaks of hemorrhagic fever in Africa. J Virol. 2004;78:8922–6. 10.1128/JVI.78.16.8922-8926.2004 15280501 PMC479050

[14] Briese T, Bird B, Kapoor V, Nichol ST, Lipkin WI. Batai and Ngari viruses: M segment reassortment and association with severe febrile disease outbreaks in East Africa. J Virol. 2006;80:5627–30. 10.1128/JVI.02448-05 16699043 PMC1472162

[15] Rodrigues DSG, Medeiros DB, Rodrigues SG, Martins LC, de Lima CP, de Oliveira LF, et al. Pacui virus, Rio Preto da Eva virus, and Tapirape virus, three distinct viruses within the family Bunyaviridae. Genome Announc. 2014;2:1e00923-14. 10.1128/genomeA.00923-14 PMC4241653 25395627

