# Supplementary material for: Detection of Novel Orthobunyavirus Reassortants in Fatal Neurologic Case in Horse and Culicoides Biting Midges, South Africa
Source: Emerg Infect Dis. 2025 Jul;31(7):1455–9. doi: 10.3201/eid3107.241800 (PMC12205456; doi:10.3201/eid3107.241800)
Supplement: Appendix 1 — Additional information for detection of novel orthobunyavirus reassortants in fatal neurologic case in horse and Culicoides biting midges, South Africa. [file 24-1800-Techapp-s1.pdf]

Article DOI: https://doi.org/10.3201/eid3107.241800

EID cannot ensure accessibility for supplementary materials supplied by authors. Readers who have difficulty accessing supplementary content should contact the authors for assistance.

Detection of Novel Orthobunyavirus Reassortants in Fatal Neurologic Case in Horse and Culicoides Biting Midges, South Africa

Appendix 1

Appendix 1 Table 1. Details of the primers, position, and target sizes used to amplify partial S, M, and L segments*

Segment	PCR round	Primer name	Position	Oligonucleotide sequence, 5′ to 3′	Target size, bp	Reference
Simbu serogroup TaqMan real-time RT-PCR						
Small	NA	Orthob1 (SimbuF152)	152–304	TAGAGTCTTCTTCCTCAAYCAGAAGAAGGCC	152	(*1*)
		Orthob1 (SimbuR304)		GTYAMGGCAMTGTCTGGCACAGGATTTG		
		Orthob1 (Simbu-probe252)		TGGTTAATAACCATTTTCC		
Simbu serogroup conventional nested PCR assay						
Small	First	Ortho1 (SimbuF141)	141–781	CGRTRYYGYTAGAGTCTTCTTCC	576	(*1*)
		Ortho1 (SimbuR718)		CGAATTGGGCAAGGAAAGT		
	Second	Ortho1 (SimbuFN403)	403–695	CCNCTTGCTGARGTNAARG	291	
		Ortho1 (SimbuRN695)		GCAGCWGGWGAGAATCCWGA		
Simbu serogroup conventional hemi-nested PCR assay						
Medium	First	SimbuM2671+	2671–3476	CGRTRYYGYTAGAGTCTTCTTCC	807	(*1*)
		SimbuM3476-		CGAATTGGGCAAGGAAAGT		
	Second	SimbuM2671+	2671–3110	CCNCTTGCTGARGTNAARG	421	
		SimbuM3110-		GCAGCWGGWGAGAATCCWGA		
		Ortho1 (SimbuRN695)		GCAGCWGGWGAGAATCCWGA		
Pan-orthobunyavirus hemi-nested PCR assay						
Large	First	Peri-F1	2907–3427	CAAARAACAGCAAAAGAYAGRGARA	513	(*2*)
		Peri-R1		TTCAAATTCCCYTGIARCCARTT		
	Second	Peri-F2	3018–3427	ATGATTAGYAGRCCDGGHGA	402	
		Peri-R2		TTCAAATTCCCYTGIARCCARTT		

*NA, not applicable; RT-PCR, reverse transcription PCR.

Appendix 1 Table 2. Positive cases identified by orthobunyavirus reverse transcription PCR of the S segment*

ZRU no.	Date received	Province	Species	Sex	Age	Specimen used for PCR	Fatality	Main syndrome	Additional information	Virus, confirmed by sequencing
ZRU053/20	2020/03/12	Mpumalanga	Ovine	M	Fetus	RNA from brain, spleen	Yes	Abortion/stillbirth,	Hydranencephaly, no brain development	Shuni virus
ZRU012/21	2021/02/08	Gauteng	Equine	M	17 y	Brain	Yes	Ataxia, head tilt	Fever 38.0°C	Shuni virus
ZRU027/21/2	2021/04/01	Gauteng	Equine	M	6 years	EDTA blood	Yes	Ataxia, head tilt	Previously positive for Sindbis virus	Shuni virus
ZRU093/21	2021/05/13	Western Cape	Caprine	M	Fully developed fetus	Brain, lung, liver, spleen	Yes	Abortion	5 of 50 ewes aborted in 2 weeks' time	Shamonda virus
ZRU019/22	2022/02/23	Northern Cape	Horse	M	5 months	EDTA blood	No	Ataxia	Found ataxic in paddock	Shuni virus
ZRU099/22	2022/06/09	North West	Ovine	M	Fetus	Lung, spleen	Yes	Abortion	Stillbirth, fetal deformity	Shuni virus

*SHAV, Shamonda virus; SHUV, Shuni virus.

References

1. Van der Walt M, Rakaki ME, MacIntyre C, Mendes A, Junglen S, Theron C, et al. Identification and molecular characterization of Shamonda virus in an aborted goat fetus in South Africa. Pathogens. 2023;12:1100. PubMed https://doi.org/10.3390/pathogens12091100

2. Kopp A, Hübner A, Zirkel F, Hobelsberger D, Estrada A, Jordan I, et al. Detection of two highly diverse peribunyaviruses in mosquitoes from Palenque, Mexico. Viruses. 2019;11:832. PubMed https://doi.org/10.3390/v11090832

3. Kumar S, Stecher G, Li M, Knyaz C, Tamura K. MEGA X: molecular evolutionary genetics analysis across computing platforms. Mol Biol Evol. 2018;35:1547–9. PubMed https://doi.org/10.1093/molbev/msy096
